# HDAC4 in ischemic stroke: mechanisms and therapeutic potential

**DOI:** 10.1186/s13148-018-0549-1

**Published:** 2018-09-12

**Authors:** Qingsheng Kong, Yongnan Hao, Xin Li, Xin Wang, Bingyuan Ji, Yili Wu

**Affiliations:** 1 Collaborative Innovation Center for Birth Defect Research and Transformation of Shandong Province, Jining Medical University, 133 Hehua Road, Taibaihu New District, Jining, 272067 Shandong China; 2 Department of Biochemistry, Jining Medical University, 133 Hehua Road, Taibaihu New District, Jining, 272067 Shandong China; 3 Department of Neurology, Affiliated Hospital of Jining Medical University, 89 Guhuai Road, Jining, Jining, 272000 Shandong China; 4 TEDA international Cardiovascular Hospital, 61 3rd AV, TEDA, Tianjin, China; 5 Institute of Mental Health, Jining Medical University, 133 Hehua Road, Taibaihu New District, Jining, 272067 Shandong China; 6 Department of Psychiatry, Jining Medical University, 133 Hehua Road, Taibaihu New District, Jining, 272067 Shandong China

**Keywords:** HDAC4, Ischemic stroke, Cell death, Angiogenesis, Neurogenesis

## Abstract

Stroke is one of the leading causes of death and disability worldwide, and the majority of the cases are ischemic stroke. However, it still lacks effective treatment except for thrombolytic therapy in an extremely narrow time window. Increased evidence suggests that histone deacetylase 4 (HDAC4) was dysregulated in ischemic stroke, which plays a key role in the pathogenesis of ischemic stroke and post-stroke recovery by affecting neuronal death, angiogenesis, and neurogenesis. Therefore, we aim to review the dysregulation of HDAC4 in ischemic stroke and the role of dysregulated HDAC4 in the pathogenesis of ischemic stroke. Furthermore, the therapeutic potential of modulating HDAC4 in ischemic stroke is discussed.

## Background

Stroke is one of the leading causes of death and disability worldwide [1]. In the USA, it is the leading cause of long-term disability, including both physical and cognitive deficits, while it is the leading cause of death in China [2, 3]. The prevalence of stroke continues increasing, and the direct medical costs will reach $184.1 billion in the USA by 2030. In addition, increased risk of neurodegenerative diseases, such as Alzheimer’s disease, was observed in patients who experienced a stroke, which further increases the burden of health care [4]. Ischemic stroke is the major subtype of stroke, accounting for 87% of stroke cases. However, current treatments for ischemic stroke are only limited to thrombolytic therapy within an extremely narrow time window [5]. Thus, developing novel therapeutic approaches for ischemic stroke is urgent.

Histone deacetylases (HDACs) along with histone acetyltransferases (HATs) regulate chromatin remodeling and subsequent gene transcription by controlling the status of histone acetylation. Compared with histone acetylation, histone deacetylation induces a condensed chromatin conformation, contributing to the repression of gene transcription which is involved in diverse physiological processes. Moreover, the function of HDACs is not limited to the histone deacetylation. Recent evidence suggests that HDACs may also contribute to the deacetylation of non-histone proteins [6]. In addition, HDACs also have deacetylase-independent functions, including other modifications of histone, such as methylation [6–8]. Importantly, HDACs are dysregulated in a number of brain disorders, which is implicated in the pathogenesis of these diseases, e.g., ischemic stroke, autism, Alzheimer’s disease, and depressive disorders [9–15]. It suggests that HDACs might be potential targets for the treatment of brain disorders.

Growing evidence indicates that HDAC4 is a specific target for the treatment of ischemic stroke. First, dysregulated HDAC4 was observed in ischemic stroke, which does play a key role in the pathogenesis of ischemic stroke and post-stroke recovery by affecting neuronal death, angiogenesis, and neurogenesis [16–20]. For example, HDAC4 is reduced in ischemic stroke model animals and oxygen-glucose deprivation (OGD)-treated neurons, while increased HDAC4 expression reduces infarct volume in ischemic stroke model animals and increases cell viability of OGD-treated neuronal cells [9, 10, 21, 22]. In addition, HDAC4 has a significant effect on a cognitive function which could be impaired by ischemic stroke [23]. For example, conditional deletion of HDAC4 leads to learning and memory deficits [24–26]. It indicates that HDAC4 might be a target for the treatment of ischemic stroke. Therefore, we aim to review the dysregulation of HDAC4 in ischemic stroke and the role of HDAC4 in the pathogenesis of ischemic stroke and post-stroke recovery. Furthermore, the therapeutic potential of modulating HDAC4 in ischemic stroke is discussed.

## Mechanisms of ischemic stroke and post-stroke recovery

### Cell death and synaptic impairment

Depending on the severity of reduced blood supply, acute and delayed cell death, i.e., necrosis and apoptosis, occurs in the core region and penumbra region of the ischemic territory, respectively [27]. Necrosis occurs within minutes after stroke, which cannot be rescued. However, apoptosis and impaired synaptic function in the penumbra could be salvageable by proper interventions, suggesting that preventing apoptosis and recovering synaptic function in the penumbra region may be an effective approach to improve post-stroke recovery. Ischemia/reperfusion injury-induced apoptosis and synaptic impairment in the penumbra are mediated by a number of mechanisms, including excitotoxicity, oxidative stress, inflammatory response, and endoplasmic reticulum (ER) stress [28–30]. For example, the dysregulation of synaptic proteins, e.g., subunits of *N*-methyl-d-aspartic acid (NMDA) receptors, was observed in ischemic stroke, which not only led to synaptic dysfunction but also contributed to excitotoxic cell death [30]. It suggests that suppressing detrimental pathways may have therapeutic potential for ischemic stroke by protecting the penumbra from neuronal death and synaptic impairment.

### Angiogenesis

During an acute ischemic stroke, the reduction of blood supply in the ischemic area often activates angiogenesis, a neurovascular remodeling process, which is a compensatory response to the reduction of oxygen. Numerous studies have shown that angiogenesis is positively correlated with the survival rate of patients who experienced an ischemic stroke, indicating that angiogenesis is an endogenous brain repair mechanism [31–33]. Thus, the modulation of vascular growth in the ischemic area could be a therapeutic approach for ischemic stroke. Indeed, the beneficial effects of direct injections or gene transfer of angiogenic factors have been demonstrated by inducing therapeutic angiogenesis in ischemic stroke, myocardial infarction, and limb ischemic injury [34–36]. Enhanced angiogenesis is not only beneficial to the cell survival in the penumbra region but also promotes neurogenesis facilitating post-stroke recovery, which orchestrates post-stroke recovery [37].

### Neurogenesis

Neurogenesis, including neural stem cell proliferation, migration, and differentiation, plays a key role in the chronic stage of post-stroke recovery [38]. Increased stem cell proliferation was observed in post-stroke patients and mice model. However, the majority of newly born cells die during the first 2 weeks after their formation. It suggests that improving the survival, migration, and differentiation of newly formed cells is the key of enhancing post-stroke neurogenesis. In addition, repetitive transcranial magnetic stimulation ameliorates cognitive impairment by enhancing neurogenesis in rats with ischemic stroke [39]. Moreover, the consistent efficacy of two approaches, stem cell transplantation and stimulating endogenous neurogenesis, was observed in animal models of ischemic stroke [40, 41]. However, the therapeutic effect of transplantation of stem cell for ischemic stroke needs to be further investigated, and clinical trials are still ongoing [40–42].

## Characteristics of HDAC4

HDACs are a large family of enzymes, regulating chromatin remodeling and subsequent gene transcription mainly by controlling the status of histone acetylation. According to the sequence homology, HDACs are grouped into class I (HDAC1, 2, 3, and 8), class II (IIa: HDAC4, 5, 7, and 9; IIb: HDAC6 and 10), class III (SIRT1–7), and class IV (HDAC11). The HDAC4 protein consists of a long N-terminal domain and a highly conserved C-terminal catalytic domain [15]. Compared with most of HDACs, HDAC4 is usually trapped in the cytoplasm. Its shuttling between the cytoplasm and nucleus is tightly controlled by both the phosphorylation status of HDAC4 and its interacting partners, such as calcium/calmodulin-dependent kinase II (CaMKII), protein phosphotase 2A (PP2A), protein kinase C (PKC), and tyrosine 3-monooxygenase/tryptophan 5-monooxygenase activation protein (14-3-3) [43–46]. For example, HDAC4 is the substrate of CaMKII, which can export HDAC4 to the cytoplasm [47].

Compared with other HDACs, HDAC4 per se features weak histone deacetylase activity. It may also contribute to the histone deacetylation via interacting with HDAC3 and HDAC5, respectively [48–50]. Moreover, HDAC4 does have histone deacetylase-independent functions. For example, HDAC4 is involved in histone methylation contributing to the regulation of gene transcription [8]. In addition, HDAC4 could regulate gene transcription by interacting with multiple transcriptional factors, including runt-related transcription factor 2 (Runx2), myocyte enhancer factor 2 (MEF2), serum response factor (SRF), heterochromatin protein 1(HP1), nuclear factor kappa B (NF-κB), and activating transcription factor 4 (ATF4). [51–53]. Furthermore, HDAC4 is implicated in regulating protein SUMOylation by interacting with the SUMO-conjugating enzyme Ubc9 (Ubc9) [54]. Thus, HDAC4 may contribute to a number of physiological and pathological processes via histone deacetylase-dependent and deacetylase-independent pathways [51, 52].

## Dysregulation of HDAC4 in ischemic stroke

HDAC4 is highly expressed in the brain, mainly in neurons [9]. Recent studies indicate that HDAC4 is dysregulated in ischemic stroke, which may play a pivotal role in the pathogenesis of ischemic stroke and post-stroke recovery. Compared with sham treatment, middle cerebral artery occlusion (MCAO)/reperfusion significantly reduces the expression of HDAC4 in the cortex of rats, which is mediated by NADPH oxidase [9, 10]. Consistently, the HDAC4 expression is significantly reduced in the cardiomyocytes following ischemia/reperfusion injury [55]. However, the expression of HDAC4 is increased in oligodendrocyte progenitor cells in the brains of ischemic stroke model rats [56].

A number of microRNAs targeting HDAC4 were altered in ischemic stroke, which may also contribute to the dysregulation of HDAC4 in ischemic stroke. For example, miR-9 and miR-124 are markedly increased in both serum and CSF of patients with ischemic stroke [57, 58]. However, Liu et al. showed that serum miR-124 and miR-9 were reduced in patients with ischemic stroke, although the sample size was small [59]. In addition, the reduction of miR-9 was detected in the brain of ischemic stroke model mice [60]. Moreover, miR-206 and miR-29b, two microRNAs targeting HDAC4, are significantly increased in ischemic rat brains and in OGD-treated primary neurons [61–63]. It suggests that the combination effect of dysregulated microRNAs may contribute to the reduction of HDAC4 in ischemic stroke.

In addition to HDAC4 expression, nuclear shuttling of HDAC4 is altered in ischemic stroke, which plays an important role in the pathogenesis of stroke and post-stroke recovery. Increased HDAC4 nuclear shuttling was observed in the neurons of ischemic stroke model mice/ats and in oxygen-glucose deprivation (OGD)-treated neurons, while the overexpression of calcium/calmodulin-dependent protein kinase IV (CaMKIV) reduced the levels of nuclear HDAC4 in ischemic stroke [11, 64]. However, increased cytoplasmic HDAC4 expression was detected in oligodendrocyte progenitor cells in the brains of ischemic stroke model rats [56].

## The role of HDAC4 in ischemic stroke and underlying mechanisms

### HDAC4 in neuronal death and synaptic impairment

**Fig. 1:**
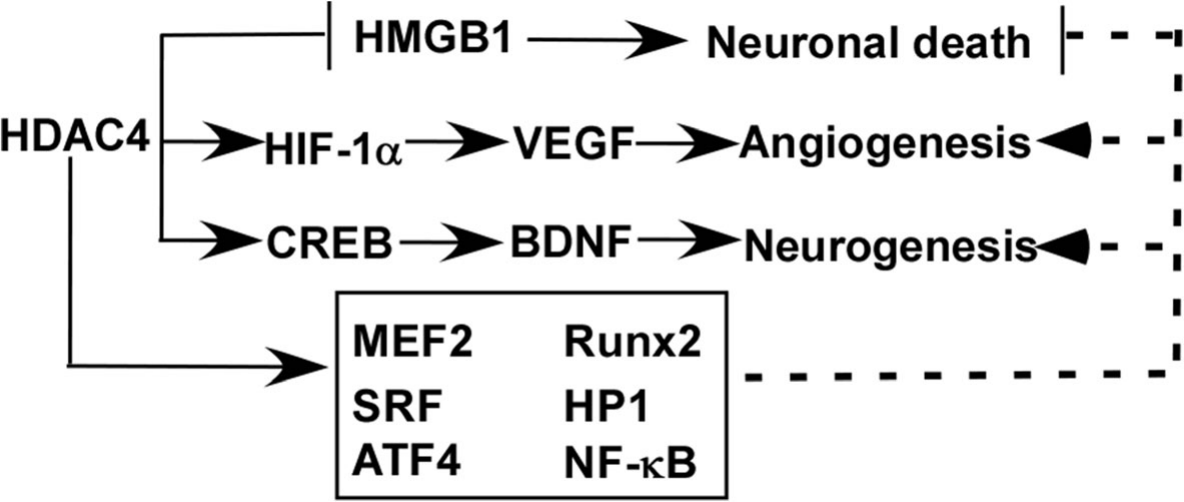
The role of HDAC4 in ischemic stroke and underlying mechanisms. HDAC4 could inhibit neuronal death via reducing HDMGB1expression and release, while it promotes angiogenesis and neurogenesis via HIF-1α-VEGF signaling and CREB-BDBF signaling, respectively. The interacting partners of HDAC4, MEF2, Runx2, SRF, HP1, ATF4, F-κB, etc. might also mediate its role in the neuronal death, angiogenesis, and neurogenesis in ischemic stroke. The solid line represents known mechanisms, while the dash line represents possible mechanisms

Accumulated evidence indicates that HDAC4 plays an important role in the post-stroke recovery by modulating neuronal death and synaptic plasticity (Fig. 1). First, HDAC4 deficiency causes a progressive loss of neurons in the cerebellum of mice, while the forcing expression of HDAC4 protects neurons from cell death [16]. Moreover, the HDAC4-C-terminal fragment is crucial to rescue HDAC4 knockdown-induced cell death and a reduction of synaptic strength in mouse brains [51]. Zhang et al. showed that reduced HDAC4 expression is associated with blood-brain barrier (BBB) breakdown contributing to ischemia/reperfusion injury-induced infarct in ischemic stroke model rats, while increased HDAC4 expression ameliorates BBB injury, contributing to the reduced infarct volume [10]. Consistently, class IIa histone deacetylase-specific inhibitor increases mortality and infarct volume in the brains of ischemic stroke model rats and exacerbates neuronal remodeling impairment, such as reduced dendritic and axonal and myelination densities [65]. However, pan-HDAC inhibitors have a protective effect on stroke [66, 67]. Moreover, HDAC4 increases cell viability of OGD-treated cells via reducing high-mobility group protein 1(HMGB1) expression [9]. In addition, a proteomics analysis indicated that HDAC4 is a regulator of proteins involved in neuronal excitability and synaptic plasticity [68]. Silencing HDAC4 expression results in the impairment of synaptic plasticity and learning and memory deficits in both mice and Drosophila, although one report showed that HDAC4 knockdown with siRNA improved the survival of OGD-treated neurons [11, 26, 69]. Currently, mechanisms of reduced HDAC4 in ischemia/reperfusion injury-induced neuronal death and synaptic impairment remain elusive. However, a number of studies indicate that the effect of HDAC4 on neuronal death and synaptic impairment might be mediated by its partners, e.g., Runx2, MEF2, SRF, HP1, NF-κB, and ATF4, contributing to the processes of ER stress, inflammation, and oxidative stress response [16, 51–53, 70, 71]. For example, HDAC4 overexpression causes ATF4 retention in the cytoplasm, inhibiting ER stress-induced apoptosis, while HDAC4 reduction exacerbates ER stress-induced apoptosis [53].

In addition to HDAC4 levels, nuclear shuttling of HDAC4 also contributes to neuronal death and synaptic impairment in ischemic stroke. Nuclear HDAC4 represses the expression of constituents of synapses leading to the impairment of synaptic architecture and strength in mice [51]. In addition, the neuroprotective effect of CaMKIV on OGD neurons is mediated by reducing nuclear HDAC4 [11]. Moreover, mice carrying nuclear HDAC4 mutant exhibit deficits in neurotransmission, learning, and memory [51]. Yuan et al. reported that ischemic stroke-induced nuclear shuttling of HDAC4 strongly facilitated OGD-induced neuronal death and exacerbated infarct volume and functional deficits in ischemic model mice [11]. In addition, accumulation of nuclear HDAC4 exerts neurotoxicity in models of Parkinson’s disease [72].

### HDAC4 in angiogenesis

Post-stroke angiogenesis has a beneficial effect on cell survival and stroke recovery. Qian et al. reported that siRNA-induced HDAC4 reduction suppressed hypoxia-inducible factor-1α (HIF-1α) expression, which inhibited HIF-1α-associated vascular endothelial growth factor (VEGF) expression in ischemia/reperfusion injury [73, 74]. It suggests that HDAC4 alteration may regulate the angiogenesis in ischemic stroke via HIF-1α-VEGF signaling (Fig. 1). Moreover, HDAC4 phosphorylation is also the key regulator of angiogenesis. Phosphorylation of HDAC4 is remarkably upregulated in the endothelial cells under hypoxic conditions while blocking the phosphorylation of HDAC4 inhibits endothelial cell migration and tube formation, which is associated with the suppression of HIF-1α-VEGF signaling [75]. Consistently, Liu et al. showed that phosphorylation of HDAC4 was associated with the induction of HIF-1α-VEGF signaling, promoting angiogenesis in ischemic stroke model mice and cells [75]. GO6976, an inhibitor of HDAC4, blocks the phosphorylation of HDAC4 and inhibits the tube formation and migration of endothelial cells [75]. It suggests that HDAC4 phosphorylation facilitates angiogenesis in ischemic stroke. Moreover, HDAC4 may be involved in angiogenesis via its interacting partners, such as NF-κB [76]. Furthermore, Madelaine et al. identified miR-9 inhibition as a positive regulator of neurogenesis and angiogenesis [77]. As HDAC4 is a target of miR-9, it may contribute to the effect of miRNA-9 inhibition on angiogenesis and neurogenesis, suggesting that HDAC4 might be a potential target for the treatment of ischemic stroke.

### HDAC4 in neurogenesis

Growing evidence indicates that HDAC4 may contribute to neurogenesis via regulating the expression and function of multiple molecules. First, HDAC4 regulates the activity and expression of cAMP response element-binding protein (CREB) and brain-derived neurotrophic factor (BDNF), respectively, which play a key role in neurogenesis after ischemic stroke [18–20, 78] (Fig. 1). For example, increased CREB activity and BDNF expression promote post-ischemic stroke neurogenesis and neuroregeneration in rats. However, nuclear shuttling of HDAC4 suppresses the transcriptional activity of CREB by reducing the interaction among acetyltransferase, CBP, and CREB, leading to the reduction of BDNF. [18–20]. In addition, HDAC4 might be another key mediator of the effect of miRNA-9 on neurogenesis in ischemic stroke as HDAC4 is the target of miRNA-9 [77, 79]. Moreover, HDAC4 may be implicated in neurogenesis by regulating the activity of its partners, such as Runx2, MEF2, SRF, HP1, NF-κB, and ATF4. For example, MEF2 promotes neurogenesis while nuclear HDAC4 suppresses the activity of MEF2 [79–81]. The above evidence suggests that the alteration of HDAC4 expression and nuclear shuttling in ischemic stroke may play a pivotal role in post-stroke recovery by affecting neurogenesis.

## Clinical perspectives

### HDAC4, a unique target for ischemic stroke treatment

HDAC4 is a unique target for the treatment of ischemic stroke compared with other HDACs, such as HDAC2 [16–20]. For example, HDAC4 features different characteristics and plays an opposite role in ischemic stroke compared with HDAC2 (Table 1). HDAC4 and HDAC2 genes are located at chromosome 2q37 and chromosome 6q21, respectively, encoding1084 and 488 amino acids, respectively. HDAC4 contains both intrinsic nuclear localization signal and nuclear export signal, while HDAC2 only contains a nuclear localization signal [82–84]. Thus, HDAC2 is mainly localized in the nucleus, while HDAC4 enriches in the cytoplasm and shuttles between the cytoplasm and nucleus [82–84]. Compared with HDAC2, HDAC4 per se features weak histone deacetylase activity as the critical tyrosine residue within the catalytic domain is substituted by histidine [85]. Compared with HDAC2, HDAC4 interacts with multiple partners, e.g., Runx, MEF2, SRF, HP1, NF-κB, 14-3-3, and Ubc9 [16, 51–53, 70, 71]. HDAC4’s partners may mediate HDAC4’ function in ischemic stroke as the partners are involved in the key processes of ischemic stroke, i.e., neuronal death, angiogenesis, and neurogenesis [53, 76, 79–81] (Fig. 1). Conditional deletion of HDAC4 leads to learning and memory deficits, while global HDACs inhibitors or HDAC2 reduction significantly improves learning and memory function in mice [24–26]. Importantly, reduced HDAC4 expression and increased nuclear shuttling are detected in ischemic stroke model cells and animals, while multiple HDACs, including HDAC2, are increased in ischemic stroke models [9–11, 17, 21, 64]. Moreover, increased HDAC4 expression reduces infarct volume in ischemic stroke model animals and increases cell viability of OGD-treated neurons, while reduced HDAC2 expression promotes neuronal survival and functional recovery in ischemic stroke model animals [9, 10, 21, 22]. Consistently, pan-HDACs inhibitors and the specific inhibitor of class І HDACs, including HDAC2, alleviate stroke-induced neurological deficits facilitating post-stroke recovery in mice. However, the specific class IIa inhibitor increases mortality and infarct volume in the brains of ischemic stroke model rats, exacerbates neuronal remodeling impairment, and has no rescue effect on neurological deficits [21, 22, 65].

**Table 1:** Difference between HDAC4 and HDAC2

	HDAC4	HDAC2
Features		
Gene locus (chromosome)	2q37	6q21
Number of amino acids	1084	488
Nuclear localization signal	+	+
Nuclear export signal	+	–
Subcellular distribution	Cytoplasm/nucleus	Nucleus
Histone deacetylase activity	Weak	Strong
Effect on cognitive function	Beneficial	Impaired
Ischemic stroke		
Altered expression	Reduced	Increased
Altered distribution	Increased nuclear shuttling	–
Rescue effect of class-specific inhibitor on neurological deficits	–	+
Effect on infarct size	Reduced	Increased

The alteration and function of HDAC4 are opposite to those of HDAC2 in ischemic stroke models, indicating that increasing HDAC4 expression is a unique target for the treatment of ischemic stroke compared with inhibiting HDAC2 and other HDACs to treat ischemic stroke. Although it is inconclusive that increasing HDAC4 expression could offer a better ischemic stroke therapy compared with HDAC2 inhibition, co-regulating HDAC4 and HDAC2 or other HDACs might have better therapeutic potential. The combination effect of increasing the HDAC4 level and inhibiting the activity of HDAC2 or other HDACs needs to be further investigated.

### Current status of HDACs-based treatment

Currently, thrombolysis with tissue plasminogen activator remains the only globally approved treatment for ischemic stroke [5]. No HDAC-based approach or agent has been approved for ischemic stroke treatment, although four pan-HDAC inhibitors, vorinostat, romidepsin, belinostat, and panobinostat, are approved by the US FDA for the treatment of cutaneous T cell lymphoma, peripheral T cell lymphoma, and multiple myeloma, respectively [86, 87]. More than 350 clinical trials involving HDAC inhibitors (https://www.clinicaltrials.gov/) have been carried out or are ongoing against various diseases, including cancers, Alzheimer’s disease, schizophrenia, asthma, and chronic obstructive pulmonary disease (COPD). However, no HDAC-based clinical trial has been carried out for ischemic stroke. Moreover, it still lacks HDAC4-based preclinical studies on larger animals, although the therapeutic effect of HDAC4 has been observed in neurons, rats, and mice. Therefore, further investigation is needed before HDAC4-based clinical trials.

### Potential of HDAC4-based therapy for ischemic stroke

**Fig. 2:**
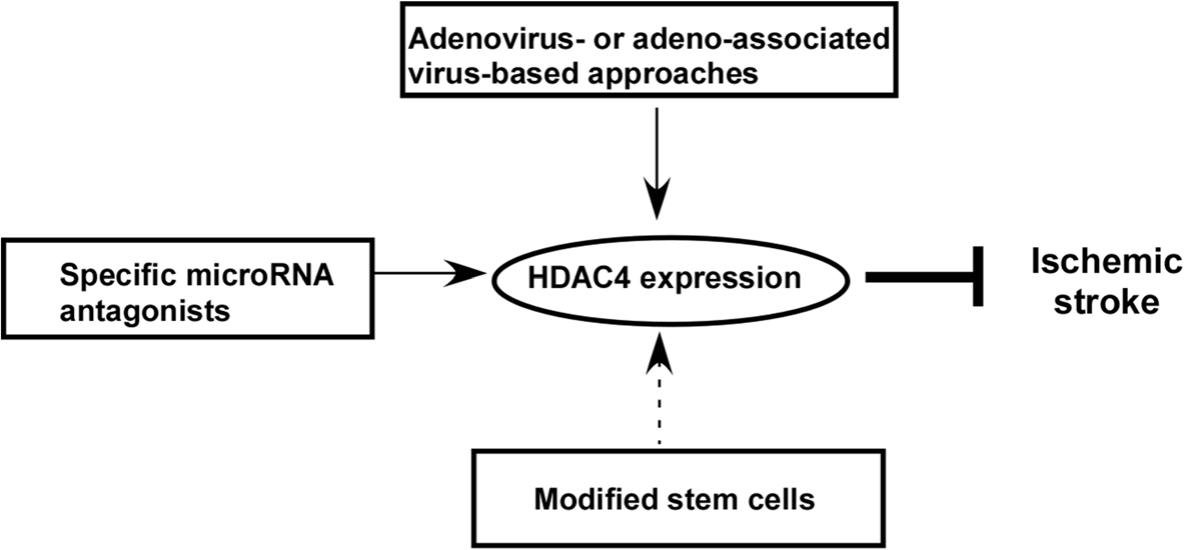
Potential of HDAC4-based therapy for ischemic stroke. Adenovirus- or adeno-associated virus-mediated HDAC4 overexpression and microRNA-based upregulation of HDAC4 have the potential to be translated into the clinic for ischemic stroke treatment. The therapeutic potential of HDAC4-modified stem cells remains elusive

Accumulated evidence suggested that increasing HDAC4 expression may have therapeutic potential for ischemic stroke treatment. Several approaches of regulating HDAC4 level could be translated into the clinic (Fig. 2). Adenovirus- and adeno-associated virus-mediated HDAC4 overexpression has been applied in vitro and in vivo, indicating that virus-based HDAC4 overexpression could be a potential gene therapy for ischemic stroke treatment [16, 88–91]. However, further preclinical investigation is needed to determine the therapeutic effect on larger animals other than rodents. In addition, the efficacy and safety need to be evaluated.

MicroRNA-based therapies hold great promise in various diseases. Significant alteration of microRNAs targeting HDAC4 has been detected in ischemic stroke patients and model animals, indicating that modulating microRNAs targeting HDAC4 could be a therapeutic approach. A number of microRNAs targeting HDAC4 are increased, e.g., miR-9, miR-124, miR-29b, and miR-206, suggesting that restoring or downregulating their levels may subsequently increase HDAC4 expression. Specific microRNA antagonists, including anti-miRs, locked nucleic acids, and antagomirs, could restore HDAC4 expression or increase HDAC4 expressions. Among them, antagomirs can be delivered without any vector or vehicle assistance. A recent study showed that intranasal administration of an antagomir specifically targeting miR-206 significantly improved memory function in the model mice of Alzheimer’s disease [92]. It suggested that the non-invasive intranasal administration of specific antagomirs could be an effective approach to increase HDAC4 expression for ischemic stroke treatment. Further preclinical investigation needs to be done to determine the specificity, efficacy, and safety of this approach. The combination effect of targeting various microRNAs needs to be investigated.

Both preclinical studies and clinical trials indicated that stem cell-based therapies would be an effective approach for the treatment of many kinds of diseases, including ischemic stroke [93, 94]. In addition to numerous preclinical studies, a variety of stem cell-based clinical trials for the treatment of ischemic stroke have been carried out or are ongoing, including neural stem cells, mesenchymal stem cells, embryonic stem cells, and induced pluripotent stem cells (https://www.clinicaltrials.gov/). For example, the consistent efficacy of neural stem cell transplantation for ischemic stroke treatment was observed in both preclinical studies and clinical trials, e.g., the trial of Pilot Investigation of Human Neural Stem Cells in Chronic Ischemic Stroke Patients (PISCES) [40, 41]. The results of the PISCES trial might be more conclusive with the enrolment of additional patients and the introduction of a placebo control group in the phase 2 trial (NCT02117635) [40]. Whether HDAC4-modified stem cells could have a better therapeutic effect in patients with ischemic stroke needs to be further investigated. First, the alteration of HDAC4 in different types of stem cells is unclear as only one report showed that both total HDAC4 and cytoplasmic HDAC4 was increased in oligodendrocyte progenitor cells of ischemic stroke model rats [56]. In addition, the role of HDAC4 in different types of stem cells and underlying mechanisms remain elusive.

## Conclusions

HDAC4 expression was reduced in ischemic stroke, which may contribute to the pathogenesis of ischemic stroke by promoting neuronal death and inhibiting angiogenesis and neurogenesis. The increased HDAC4 expression could inhibit neuronal death via reducing HMGB1 expression and release and promote angiogenesis and neurogenesis via HIF-1α-VEGF signaling and CREB-BDBF signaling, respectively. The interacting partners of HDAC4, MEF2, Runx2, SRF, HP1, ATF4, and NF-κB might also mediate its role in inhibiting neuronal death and promoting angiogenesis and neurogenesis in ischemic stroke. Importantly, it remains to find similar pattern and mechanisms in patients with ischemic stroke as most studies are performed in cultured neurons and animal models. Currently, a number of approaches to regulate HDAC4 level have the potential to be translated into the clinic, such as adenovirus-/adeno-associated virus-mediated HDAC4 overexpression and microRNA-based upregulation of HDAC4. Although a variety of stem cell-based clinical trials for the treatment of ischemic stroke has been carried out or are ongoing, the therapeutic potential of HDAC4-modified stem cells remains elusive. Therefore, modulating HDAC4 expression could be translated into the clinic as an effective treatment for ischemic stroke. However, the therapeutic potential of HDAC4-modified stem cells needs to be further investigated in preclinical studies.

## Abbreviations

NF-κB: Nuclear factor kappa B
14-3-3: Andtyrosine 3-monooxygenase/tryptophan 5-monooxygenase activation protein
BBB: Blood-brain barrier
BDNF: Brain-derived neurotrophic factor
CaMK IV: Calcium/calmodulin-dependent protein kinase IV
CaMKII: Calcium/calmodulin-dependent kinase II
CREB: cAMP response element-binding protein
CSF: Cerebrospinal fluid
ER: Endoplasmic reticulum
HDAC: Histone deacetylase
HP1: Heterochromatin protein 1
MCAO: Middle cerebral artery occlusion
MEF2: Myocyte enhancer factor 2
NADPH: Nicotinamide adenine dinucleotide phosphate-oxidase
NMDA: *N*-methyl-d-aspartic acid
OGD: Oxygen-glucose deprivation
PKC: Protein kinase C
PP2A: Protein phosphotase 2A
Runx2: Runt-related transcription factor 2
SIRT1: Sirtuin 1
SRF: Serum response factor
